# Development of an ICF Core Set for adults with Chagas disease: a qualitative study capturing patients’ perspective on functioning

**DOI:** 10.1590/0037-8682-0401-2025

**Published:** 2026-06-15

**Authors:** Natielle Cecilia Santos Ottone, Henrique Silveira Costa, Sara Gabrielle Souza, Paulo Victor Mendes Santos, Rafael de Oliveira Castro, Pedro Henrique Scheidt Figueiredo, Vanessa Pereira de Lima, Marcus Alessandro de Alcantara

**Affiliations:** 1 Universidade Federal dos Vales do Jequitinhonha e do Mucuri, Programa de Pós-Graduação em Reabilitação e Desempenho Funcional, Diamantina, MG, Brasil.; 2 Universidade Federal dos Vales do Jequitinhonha e do Mucuri, Departamento de Fisioterapia, Diamantina, MG, Brasil.

**Keywords:** International Classification of Functioning, Disability and Health, Validation studies, Chagas disease, Rehabilitation, Chronic disease

## Abstract

**Background:**

Chagas disease affects multiple dimensions of human functioning, requiring assessments that extend beyond the biomedical model. The International Classification of Functioning, Disability and Health (ICF) offers an integrative framework for capturing these impacts. This qualitative study aimed to identify, from the perspective of adults diagnosed with Chagas disease, the aspects of functioning most relevant to them, as a step toward developing an ICF Core Set for this population.

**Methods:**

Thirteen focus group discussions were conducted with adults diagnosed with Chagas disease, covering all ICF components. Two independent researchers extracted meaningful concepts from verbatim transcripts and linked them to ICF categories.

**Results:**

Thirty-two adults with Chagas disease participated. From the discussions, 248 concepts were identified and linked to 49 second-level ICF categories: 13 related to body functions, 5 to body structures, 18 to activities and participation, and 13 to environmental factors. The most frequently mentioned categories were sleep, pain, emotional functions, and cardiac functions. In the Activities and Participation component, limitations were noted in household tasks, mobility, and social participation, with spirituality emerging as a key coping resource. For Environmental Factors, immediate family support and access to healthcare services were identified as primary facilitators of functioning.

**Conclusion:**

Adults with Chagas disease reported impairments across a wide range of body functions and activities, underscoring the significant influence of environmental factors. The identified categories will serve as a foundation of candidate items to support the consensus process for developing an ICF Core Set for adults with Chagas disease.

## INTRODUCTION

Chagas disease is a significant parasitic infection that affects an estimated 6 to 7 million individuals worldwide and remains endemic in 21 countries across the Americas^1^. It is caused by the protozoan *Trypanosoma cruzi* and is primarily transmitted through the bite of triatomine insects, although other routes of transmission include congenital transmission, blood transfusion, and ingestion of contaminated food^2^. Following the acute phase, the infection typically progresses to a chronic phase, whereas the subsequent development of clinical manifestations varies among individuals. In the chronic phase, some individuals may develop severe cardiac complications, such as arrhythmias, heart failure, and aneurysms. They may also present with gastrointestinal manifestations, including megaesophagus and megacolon^3^. These complications substantially impair patients’ daily functioning and considerably diminish their quality of life^4^ ^-^ ^7^.

Patients with Chagas disease frequently report symptoms such as fatigue, emotional distress, and concerns related to their health condition and treatment^8^. These experiences may exacerbate symptom perception, thereby increasing the risk of complications and hospital admissions^9^. Furthermore, the disease is associated with limitations in activities and restrictions in social participation, particularly in adverse contexts such as limited access to quality healthcare services, lack of social support, and unfavorable socioeconomic conditions^10^ ^,^ ^11^.

Considering the lived experience of people with Chagas disease, their limitations often extend beyond the cardiogastric damage described in biomedical approaches. Capturing this complexity requires a biopsychosocial framework capable of articulating impairments as well as limitations in activities and participation. The International Classification of Functioning, Disability and Health (ICF) can fulfil this role by integrating the biological, individual, and environmental components of human functioning within a single taxonomy^12^.

However, the ICF contains more than 1,400 second-level categories, which makes its implementation in clinical practice and research challenging^13^. To address this challenge, the WHO advocates the development of Core Sets, which are streamlined selections of categories considered most relevant for a specific health condition. The development process includes three complementary phases: preparatory studies, a consensus conference, and validation. However, an ICF Core Set for adults with Chagas disease is not yet available.

The process of developing an ICF Core Set follows a structured, multi-perspective approach^14^. It begins with four preparatory studies aimed at capturing diverse perspectives: a qualitative study focusing on patients’ experiences, a systematic literature review representing the researcher’s viewpoint, a survey of experts reflecting health professionals’ opinions, and an empirical clinical study.

The findings from these studies are synthesized into a preliminary list of candidate ICF categories. This list is then reviewed by a panel of experts responsible for selecting the final set of categories to be included in the Core Set. Importantly, evidence from recent studies indicates that incorporating users’ perspectives into this process allows additional domains to emerge, particularly environmental factors. These factors are often underestimated by professionals, highlighting the importance of including patients’ voices from the qualitative stage onwards^15^ ^,^ ^16^.

In this qualitative study, our objective was to identify aspects of functioning, as well as environmental and personal factors, that represent the priorities arising from the lived experiences of adults with Chagas disease.

## METHODS

### Design 

We conducted a qualitative, cross-sectional study involving patients with a confirmed clinical diagnosis of Chagas disease to explore their perspectives through individual interviews. The research was conducted at the Cardiovascular Physiotherapy Laboratory, located within the Physiotherapy Clinic on the JK Campus of the Federal University of the Vales do Jequitinhonha e Mucuri. It followed ethical standards for studies involving human participants, in accordance with the Declaration of Helsinki, and received approval from the Institutional Research Ethics Committee (CAAE 79466924.8.0000.5108). Written informed consent was obtained from all participants prior to their inclusion in the study.

### Individuals

The study was conducted between February and August 2024 with adults diagnosed with Chagas disease, recruited from municipalities in the Jequitinhonha Valley, an endemic region in the state of Minas Gerais, Brazil. Diagnosis was established based on serological confirmation of *T. cruzi* infection, in accordance with current clinical guidelines.

All participants were enrolled in JequiProj: *Evaluation and Management of Patients with Chagas Disease*, a project of the Federal University of the Vales do Jequitinhonha and Mucuri (UFVJM) aimed at training healthcare professionals in the assessment and management of Chagas disease, with a focus on Primary Health Care.

Individuals were eligible if they met the following criteria: (i) confirmed diagnosis of Chagas disease according to the International Classification of Diseases, 10th Revision (ICD-10), code B57; (ii) age between 20 and 85 years; and (iii) a clinically stable condition, without functional limitations that could hinder participation in the proposed activities.

Exclusion criteria were: (i) the presence of severe psychiatric or neurological conditions that could interfere with participation; (ii) inability to understand or perform any of the study procedures; and/or (iii) difficulty engaging in group activities.

The sample size was defined based on data saturation^17^, which corresponds to the stage at which the researcher gathers sufficient information from the field^18^ (see Data analysis).

### Data Collection

Semi-structured focus groups were led by an experienced moderator, with support from an assistant, following guiding questions developed in alignment with the components of the International Classification of Functioning, Disability and Health (ICF). These questions were developed according to Selb et al.^14^ to explore patients’ perceptions of their physical and mental health, daily challenges, and factors that either facilitate or hinder their routine.

Specifically, the questions addressed: (i) Body functions, such as energy, sleep, movement, and heart function, with prompts like: “When you think about your physical and mental body, is there anything that is not working as it should?”; (ii) Body structures, identifying specific parts that cause problems, with prompts such as: “Is there any part of your body that bothers you or does not work well?”; (iii) Activities and participation, addressing difficulties in daily routines, with questions like: “What are the most difficult things for you to do in your daily life?”; (iv) Environmental factors acting as facilitators, exploring the support available within individuals’ environments, with prompts such as: “What do you find helpful or supportive in the place where you live?”; (v) Environmental factors acting as barriers, identifying obstacles in the environment, with questions like: “What in your environment makes things more difficult in your daily life?”; and (vi) Personal factors, examining beliefs, attitudes, and behaviors that influence how individuals cope with their condition, with prompts such as: “What is important about you and the way you deal with your situation?”.

The sessions were designed to encourage spontaneous discourse and to capture individuals’ perceptions of their health and the challenges they face in everyday life. Each session lasted between 18 and 42 minutes, was audio-recorded, and was transcribed word for word, allowing for the comprehensive capture of qualitative data, which was later analyzed and mapped to ICF categories.

### Data Analysis

The analysis was conducted in two stages: a qualitative analysis followed by the linking process to the *International Classification of Functioning, Disability and Health* (ICF). The qualitative analysis was carried out using the meaning condensation method^17^, which consists of segmenting the transcripts into meaning units whenever thematic shifts occur.

This phase was independently performed by two researchers, who read the transcripts in full to obtain a comprehensive understanding of the content, identified meaning units, and extracted and grouped concepts into recurring themes. The process involved three steps: (i) reading the transcripts in full; (ii) identifying meaning units, defined as text segments that share a common theme; and (iii) extracting and grouping concepts into a list of recurring themes.

The analysis was conducted using structured data extraction tables (e.g., Microsoft Excel) to organize and compare the identified concepts.

Each identified concept was then linked to ICF categories according to the standardized linking rules proposed by Cieza et al.^19^ ^,^ ^20^. Two independent reviewers (NCSO and PVMS), trained in the application of the ICF and the linking methodology, conducted this process. In cases of disagreement, consensus was adopted as the decision criterion, and when consensus could not be reached, a third researcher (MAA) was consulted.

To assess the coverage of the Comprehensive ICF Core Set for Chagas disease, the categories identified in the interviews were compared with those listed in the ICF Checklist, version 2.1a^14^. Categories emerging from the interviews and also present in the checklist were classified as “confirmed,” whereas those absent from the checklist were classified as “not confirmed.”

Data saturation was established *a priori*, following the definition proposed by Coenen et al.^21^, and was defined as the stage in data collection and analysis at which linking concepts from two consecutive focus groups no longer yielded additional relevant second-level ICF categories. This criterion, grounded in the recurrence of previously identified themes, ensured that the dataset was sufficiently comprehensive to enable an in-depth exploration of individuals’ experiences. Agreement between the two researchers in the qualitative analysis was evaluated by inter-observer reliability using Cohen’s kappa, with an overall coefficient subsequently calculated²².

## RESULTS

A total of 32 individuals with Chagas disease participated in thirteen focus groups, with a mean age of 66 ± 8 years and a predominance of women (56.2%). The focus group sessions lasted between 18 and 42 minutes, with an average duration of 28 minutes, excluding breaks.

**FIGURE 1:**
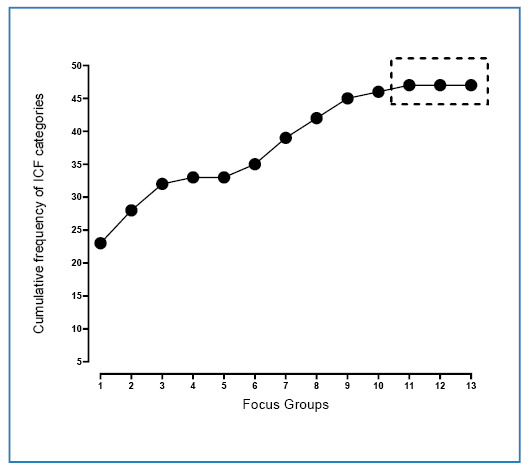
Qualitative data saturation in the focus groups. *Cumulative frequency of the categories identified from the International Classification of Functioning, Disability and Health (ICF) for the Chagas Disease Core Set.

Data saturation was achieved when the final two interviews failed to yield any new relevant second-level categories for the development of the ICF Core Set for Chagas disease (Figure 1). This criterion, based on the recurrence of previously identified themes, indicated that the information gathered was sufficiently comprehensive to enable an in-depth understanding of individuals’ experiences. The inter-observer reliability analysis resulted in an overall Cohen’s kappa coefficient of 0.51, indicating moderate agreement^22^ .

### Identified ICF Categories

The qualitative analysis of interviews with patients with Chagas disease resulted in the identification of 248 concepts, which were linked to 49 second-level ICF categories. These categories reflect the main areas in which the disease affects patients’ lives, encompassing physical, emotional, social, and environmental aspects.

Within the Body Functions component (b), a total of 97 concepts were identified. Mental functions (b1) were the most frequently reported, comprising 46.4% of the mentions, with notable impacts on areas such as sleep (b134) and emotional functions (b152). Functions related to the cardiovascular, hematological, immunological, and respiratory systems (b4) accounted for 18.6% of the concepts, particularly heart functions (b410). (Table 1).

In the Body Structures component (s), 29 concepts were identified, predominantly associated with movement-related structures (s7), which represented 65.5% of the mentions, and with structures of the cardiovascular, immunological, and respiratory systems (s4), accounting for 34.5% (Table 1).

**TABLE 1:** Participants’ concepts linked to ICF categories: Body Functions (b) and Body Structures (s).

ICF Code	ICF Category Title	n
Body Functions (n = 97)		
b130	Energy and drive functions	11
b134	Sleep	13
b140	Attention	1
b144	Memory	8
b152	Emotional functions	12
b235	Vestibular functions (including balance functions)	3
b280	Sensation of pain	13
b410	Heart functions	12
b440	Respiratory system functions (breathing)	6
b515	Digestive functions	7
b525	Defecation functions	7
b620	Urinary functions	1
b730	Muscle power functions	3
Body Structures (n = 29)		
s410	Cardiovascular system	5
s430	Respiratory system	5
s730	Upper extremity (arm, hand)	5
s750	Lower extremity (leg, foot)	9
s760	Trunk	5

The Activities and Participation component (d) comprised 66 concepts, with the highest frequencies in community, social, and civic life (d9), representing 34.8% of mentions, mobility (d4), representing 25.8%, and domestic life (d6), representing 22.7%. The category religion and spirituality (d930) was particularly relevant, accounting for 16.7% of mentions (Table 2).

**TABLE 2:** Participants’ concepts linked to ICF categories: Activities and Participation (d).

ICF Code	ICF Category Title	n
Activities and Participation (n = 66)		
d220	Undertaking multiple tasks	2
d310	Communication - receiving spoken messages	1
d430	Lifting and carrying objects	8
d440	Fine hand use (picking up, grasping)	1
d450	Walking	7
d470	Using transportation (cars, buses, trains, airplanes, etc.)	1
d510	Washing oneself (bathing, drying, washing hands, etc.)	1
d540	Dressing	1
d570	Looking after one’s health	1
d620	Acquisition of goods and services (shopping, etc.)	4
d630	Preparing meals (cooking, etc.)	2
d640	Doing housework (cleaning, washing dishes, laundry, ironing, etc.)	9
d730	Relating with strangers	1
d760	Family relationships	3
d850	Remunerative employment	1
d910	Community life	6
d920	Recreation and leisure	6
d930	Religion and spirituality	11

Regarding Environmental Factors (e), 56 concepts were identified, most frequently in support and relationships (e3), representing 46.4% of mentions, and services, systems, and policies (e5), representing 30.4%. Immediate family (e310) stood out as a crucial source of support, accounting for 21.4% of mentions (Table 3). 

**TABLE 3:** Participants’ concepts linked to ICF categories: Environmental Factors (e).

ICF Code	ICF Category Title	n
Environmental Factors (n = 56)		
e110	Products or substances for personal consumption (food, medication)	5
e120	Products and technology for personal indoor and outdoor mobility and transportation	2
e310	Immediate family	12
e320	Friends	3
e325	Acquaintances, peers, colleagues, neighbors, and community members	8
e355	Health professionals	3
e410	Individual attitudes of immediate family members	3
e420	Individual attitudes of friends	1
e450	Individual attitudes of health professionals	2
e525	Housing services, systems, and policies	4
e540	Transportation services, systems, and policies	2
e575	General social support services, systems, and policies	1
e580	Health services, systems, and policies	10

## DISCUSSION

This qualitative study revealed a wide spectrum of challenges faced by adults with Chagas disease. Individuals frequently reported difficulties with sleep and emotional functioning, along with impairments in structures associated with movement and the cardiovascular system. They also highlighted limitations in domestic and community life activities, as well as the critical role of family support and adequate access to healthcare services.

The most commonly reported issues included severe fatigue, persistent sleep disturbances, significant emotional distress, chronic pain, and clinically significant cardiovascular manifestations. These symptoms, which were often interrelated, formed a multifactorial clinical profile in which physical exhaustion, disrupted sleep, pain, and emotional suffering coexisted and mutually exacerbated one another, substantially worsening functional limitations and quality of life^7^ ^,^ ^23^ ^-^ ^25^ .

Preclinical and clinical evidence suggest that neuroinflammatory processes, persistent *T. cruzi* infection in the central nervous system, and oxidative stress may be associated with mood disturbances such as anxiety and depression, as well as cognitive deficits and sleep impairment in patients with chronic Chagas disease^26^. These findings should be interpreted with caution, as the current evidence remains limited and does not establish causal relationships. 

In parallel, cardiac dysfunction remains a hallmark of the chronic phase, underscoring the systemic nature of Chagas disease and the complexity of its clinical management^27^. This systemic involvement may help explain the interaction between cardiovascular impairment and neurological and psychosocial manifestations, including fatigue, reduced functional capacity, and emotional distress. Collectively, these findings emphasize the urgent need for integrated clinical approaches that simultaneously address cardiovascular, neurological, and psychosocial domains.

Compared with the numerous references to impairments in body functions, individuals made relatively few explicit mentions of anatomical structures, suggesting that they tended to describe their experiences in terms of psychophysiological consequences rather than specific anatomical anomalies. Among these structural categories, impairments in the lower extremities were the most prominent. Although less frequently mentioned, structural involvement of the cardiovascular system, respiratory system, trunk, and upper extremities was also noted, reflecting broader systemic manifestations. These findings may be related to the functional consequences of cardiac involvement, which lead to persistent fatigue, reduced exercise tolerance, and social withdrawal; in turn, these consequences promote a sedentary lifestyle, further exacerbating deconditioning and lower-limb muscle weakness^28^. 

With respect to the activities and participation component, the most frequently reported ICF categories included religion and spirituality, domestic activities, lifting and carrying objects, and walking. The prominence of religion and spirituality underscores their vital role as coping strategies for emotional distress, particularly in contexts of social vulnerability and chronic suffering^25^ ^,^ ^29^. Adults with Chagas disease report a substantial impact on daily activities, often hindered by fatigue, pain, and dyspnea^24^ ^,^ ^27^. Restrictions in community life, recreation, and leisure are consistent and point to reduced social participation, potentially reinforcing isolation and undermining subjective well-being^7^.

We observed a greater number of environmental factors, indicating that adults with Chagas disease perceive these elements as highly relevant, despite being relatively underexplored in the literature^30^. Support from the immediate family stood out as a key facilitator of functioning, offering both emotional and practical assistance. In contrast, health services, although essential, were often criticized because of issues related to access and quality. These findings highlight the importance of incorporating the perspectives of adults with Chagas disease to achieve a more comprehensive understanding of the factors influencing their functioning.

Some limitations of the study must be acknowledged. Individuals were recruited in the Alto Jequitinhonha region, and the findings may reflect the experiences of individuals from a specific geographic and sociocultural context. In addition, participants were recruited from among individuals receiving care at a university-based outpatient diagnostic and follow-up service. This may have favored the inclusion of those with greater access to health services or more active engagement in their treatment, possibly underrepresenting the experiences of individuals who are more socially isolated or disengaged from care.

Despite these limitations, additional perspectives will be captured in the subsequent phases of ICF Core Set development, including expert consultations and empirical studies, thereby enhancing the comprehensiveness and applicability of the final set.

This qualitative study generated an initial set of ICF categories that capture the lived experiences and perceived priorities of individuals with Chagas disease. Collectively, these categories encompass relevant aspects of functioning, disability, and environmental factors, underscoring that Chagas disease extends beyond a strictly biomedical perspective. Instead, it demands interventions that recognize psychosocial and cultural determinants of functioning and social participation, aiming not only to manage symptoms but also to sustain autonomy, community involvement, and quality of life.

## Data Availability

Research data is available in the body of the article (Results).
